# The measurement of plasma fructosamine as a diagnostic tool to improve the interpretation of plasma glucose and proteins in alpacas (*Vicugna pacos*)

**DOI:** 10.1038/s41598-024-73399-4

**Published:** 2024-09-27

**Authors:** Frederik Kiene, Johannes Buchallik-Schregel, Petra Röhrig, Saskia Neubert, Alexandra von Altrock, Benjamin U. Bauer, Thies J. Nicolaisen, Marion Schmicke, Martin Ganter, Matthias Gerhard Wagener

**Affiliations:** 1 Clinic for Swine and Small Ruminants, Forensic Medicine and Ambulatory Service, University of Veterinary Medicine Hannover, Foundation, Hannover, Germany; 2 Clinical Endocrinology Laboratory, Clinic for Cattle, University of Veterinary Medicine Hannover, Foundation, Hannover, Germany

**Keywords:** Camelids, Diabetic like syndrome, Hyperglycemia, Albumin, Cortisol, Biomarkers, Preclinical research

## Abstract

This study assesses the use of fructosamine as a diagnostic tool for hyperglycemia in alpacas in view of their sensitivity to stress and susceptibility to conditions like lipid mobilization syndrome. Plasma fructosamine, like in diagnosing diabetes in cats and dogs, can reveal long-term blood glucose trends, differentiating stress-induced spikes from persistent diabetic hyperglycemia. In 125 alpacas presented as patients of a veterinary clinic, plasma glucose and fructosamine concentrations were compared for correlations with findings of the general clinical examination, laboratory parameters, demographic data, and a behavioral stress assessment processed by using principal component analysis. Hyperglycemia was observed on admission of 71% (89/125) of the animals. This was significantly associated with a higher concentration of serum cortisol and a higher behavioral stress scoring. Fructosamine above the reference limit was detected in only 15% (13/89) of the hyperglycemic individuals. In addition to a positive correlation of fructosamine to glucose concentration, positive relationships with different plasma proteins were detected. A relationship to stress parameters was not observed. These findings underscore stress as a significant trigger for hyperglycemia in alpacas and suggest fructosamine as a valuable parameter for distinguishing between stress-induced and diabetic hyperglycemia. However, the dependence of fructosamine formation on total plasma protein concentration should be considered to avoid misinterpretation.

**Supplementary Information:**

The online version contains supplementary material available at 10.1038/s41598-024-73399-4.

## Introduction

During veterinary examination of South American camelids (SACs), which are increasingly presented as patients to veterinarians in Europe^1^, often high plasma glucose concentrations are detected^2^. If iatrogenic and dietary reasons can be excluded, hyperglycemia could either be physiologically stress-induced, related to pain and transport before and handling during the examination^3,4^, or the result of a pathological condition. Primary disorders of glucose metabolism such as diabetes mellitus might be responsible^5^. Secondary disorders caused by an intracellular energy deficiency involving the fat and energy metabolism like hyperlipemia, hepatic lipidosis, and ketosis related to a diabetic like syndrome can also be associated^2,6–8^.

Compared to other species, gluconeogenesis is assessed to be more effective in camelids^9^. Also, less insulin is secreted, and the degree of intrinsic insulin resistance is higher than in most mammals^9–13^. Due to this physiological insulin resistance in camelids, these species usually show hyperglycemia in the case of a fat metabolism disorder^2^, while ruminants in similar situations mostly show glucose concentrations below the reference interval^14^. Considering this, the possibility of differentiating physiological short-term from pathological long-term hyperglycemia in SACs is clearly relevant. So far it has only been possible with elaborate glucose tolerance testing or continuous glucose monitoring by the application of devices from human medicine^10,15^.

In veterinary medicine, the plasma fructosamine concentration was already used for many years to distinguish between diabetic and short-term stress-related hyperglycemia in cats and dogs^16,17^. Fructosamine is a ketoamine that results from random non-enzymatic linking of glucose or mannose to proteins^18,19^. Its concentration is hence directly proportional to the plasma concentration of glucose over a period of 2–3 weeks^16,20^, which might be elevated over longer periods of time in relation to a diabetic like syndrome or chronic stress. In addition to the availability of glucose, the fructosamine concentration depends on the availability of protein. This dependence can be traced specifically via the concentration of plasma albumin, as approximately 80% of ketoamines consist of glycated albumin^17,19^. However, immunoglobulins and acute-phase proteins can also contribute to the formation of fructosamine^21^. With regard to diabetes diagnostics, falsely elevated values can therefore be expected in the case of acute-phase response and falsely low values in the case of hypoproteinemia.

Beyond small animal medicine, different aspects of fructosamine as a parameter were already investigated in domestic ruminants. For cattle, fructosamine was first evaluated by Jensen et al. in 1993^22^. In this context, a reference interval was determined, the dependence of fructosamine on glucose concentration was confirmed and diurnal variations of the fructosamine concentration were excluded. In numerous other studies, fructosamine, measured both in plasma and serum, was determined as an estimator for the availability of energy in individuals in highly stressful metabolic situations such as undernutrition^23,24^ and the transition period in cattle^25–33^ as well as late pregnancy in sheep^34–36^ and in goats^37^. Other studies linked fructosamine concentration to gastrointestinal parasite infections of sheep to reflect the long-term protein loss caused by the parasites and enable the assessment of infection severity^38,39^.

Data on fructosamine determination are only scarce for alpacas and not yet available at all for other species of the camelid family: One case report on an alpaca with lipid keratopathy and atherosclerosis observed serum fructosamine concentrations of 288–409 µmol/l in four male individuals, which were assessed as normal and without any clinical relevance^40^. A publication on hematological and biochemical reference intervals in alpacas mentioned a reference interval of 251–431 µmol/l in serum and 252–425 µmol/l in plasma based on 74 healthy individuals of both sexes^41^. In a recently published study^42^, we found similar plasma concentrations of fructosamine in llamas (*Lama glama*) as described for alpacas. We also detected associations of fructosamine with sex, body condition score, and other hematological and serum parameters in llamas. Possible interactions and dependencies of fructosamine with demographic, clinical and laboratory parameters in alpacas have not yet been investigated. In this study, fructosamine is hence evaluated for the application in alpacas. Possible influences of various demographic, clinical, behavioral and laboratory diagnostic parameters on the plasma concentrations of glucose and fructosamine will be compared using a population of 125 mixed-sex alpacas, which were presented as patients to a veterinary clinic. Particular focus is placed on the comparative effects of stress on glucose and fructosamine concentrations. While fructosamine should not be affected by acute stress, a significant increase of the glucose plasma concentration is anticipated.

## Materials and methods

### Study population

The study population consisted of a total of 125 alpacas (55 intact males, 19 neutered males, 51 females), which were presented at the clinic between January 2022 and January 2023 as patients with a wide range of different symptoms and medical histories. The age of the animals ranged from three days to 18 years (6657 days) with a median age of 3.5 years (1288 days). The most common reasons for veterinary consultation were nonspecific weakness and anorexia in various gradations (26 times), acute abdominal pain (16 times), and dyspnea and diarrhea (12 times each). With a few exceptions, all animals received hospitalization. All animals were clinically examined and weighed on admission in a standardized manner. A standardized set of blood (jugular vein) and fecal samples was taken within 20 min after the clinical examination and was immediately processed for further laboratory analyses. Body condition was assessed as described in Wagener and Ganter (2020)^43^ based on the palpation of the lumbar spine and scored (BCS) with values from 1 (emaciated) in increments of 0.5 to 5 (obese). All methods were carried out in accordance with relevant guidelines and regulations and are reported in accordance with the ARRIVE guidelines.

### Behavioral scoring

Behavioral scoring to assess behavioral stress response was based on an approach published by Windschnurer et al. (2020)^44^ and was applied to 119 of the 125 individuals. The behavior of the animal on the way from the transport vehicle to the examination facility was scored in a “leading score” in ascending order from “cooperative” (score 0) to “need to be urged” (score 1) to “several attempts necessary to get the animal to stand up” (score 2) to “animal needs to be carried” (score 3). Vocalizations (“humming”, “screaming”, “snorting”, and “moaning”), defense behavior (“rising”, “spitting”, “stomping”, and “kicking”) and signs of stress (“freezing” and “collapsing”) were recorded semi-quantitatively with the scores 0 (none), 1 (1–3 times), and 2 (> 3 times). Vocalizations, defense behavior and signs of stress were only recorded for the first 20 min of clinical examination. Animals that were unable to stand for pathological reasons (10/119) were excluded from the scoring.

### Laboratory analyses

Blood samples were collected in EDTA, lithium-heparin and serum tubes (Monovette 9mL K3E, Monovette 9mL LH, Monovette 9mL Z, all from Sarstedt AG & Co. KG, Nümbrecht, Germany). Lithium-heparin and serum tubes were centrifuged at 2000 g for 15 min within 30 min of collection to separate plasma or serum. The resulting supernatant was carefully transferred to new tubes and stored at -20 °C until further analysis.

Hematological parameters including leukocyte count [10^9/l], packed cell volume (PCV) in l/l, and neutrophil-to-lymphocyte ratio (NLR) were based on EDTA samples and determined using standard manual methods as previously described by Wagener et al. (2018, 2021)^45,46^.

Fructosamine levels [µmol/l] were determined in plasma using the Cobas Mira Plus^®^ analyzer (Roche Pharma AG, Basel, Switzerland) with a commercial kit (“Fruktosamin”, Labor + Technik Eberhard Lehmann GmbH, Berlin, Germany). The method employed a colorimetric approach, where fructosamine reduces nitro blue tetrazolium to formazan in an alkaline environment. A consistent quality of the measurement was reviewed one to three times on each working day with two different control sera provided by the manufacturer. The assay was not separately evaluated for use in alpacas but widely used for the examination of samples from different taxa, including llama^42^. Information on the method for fructosamine determination in alpacas in the two previously published studies by Richter et al. (2006)^40^ and Dawson et al. (2011)^41^ is not available.

Additional biochemical parameters analyzed in plasma comprised total protein [g/l], albumin [g/l], and glucose [mmol/l]. These measurements followed the standard protocols of the clinic, as reported by Grimm et al. (2021)^47^ and Wagener et al. (2018)^48^. Globulin concentration [g/l] was calculated as the difference between total protein and albumin. Cortisol [nmol/l] in serum was measured using a chemiluminescence immunoassay (Immulite 1000 systems, Siemens Healthcare Diagnostics GmbH, Eschborn, Germany). The assay was not separately evaluated for use in alpacas. However, an identical approach was successfully applied to alpaca samples in a previous study^49^. For the interpretation of the laboratory parameters, reference intervals from Dawson et al. (2011)^41,50^ were considered.

Fecal samples were examined for parasites according to Roden (2022)^51^. However, the method was adapted to the extent that a minimum of 10 g of feces was measured accurately and used to calculate the number of eggs shed per gram of feces. In contrast to the McMaster technique, where only flotation is used, this process combines the Baermann-Wetzel larval migration method with two combined sedimentation-flotation approaches. After sedimentation with distilled water, the first approach applies saturated saline solution, mainly for the detection of strongyle type eggs; the second approach uses sodium silicate solution, mainly for the detection of liver fluke eggs. The method is applied in the clinic routine to improve the detection of parasite types such as lung worms, liver flukes, *Strongyloides papillosus*, and *Moniezia* spp. The detected number of strongyle type eggs per gram of feces in relation to the McMaster technique is, however, reduced by a factor of approximately 100^52^. Results of the shed strongyle type eggs were interpreted and semi-quantitatively scored (no, low-grade, medium-grade, high-grade, severe gastrointestinal nematode infection [GIN-Score]) according to Neubert et al. (2022)^53^.

### Statistical analyses

Statistical analyses were conducted in the R environment^54^. To compress the behavioral scoring data for further analyses in relation to the laboratory data, a principal component analysis (PCA) was implemented. The leading score data, data on vocalizations, defense behavior, signs of stress, BCS, and plasma cortisol concentration as a marker of stress were combined into superordinate principal components attributed to each individual animal. The PCA was performed using the R-command “principal()” from the package “psych”^55^. Principal components (PC1, PC2) with Eigenvalues of > 1.5 and high explanatory power (PC1: 25%, PC2: 23%) were selected as predictor variables for the further analyses. The interpretation of the effects was based on factor loadings of the principal components (Table 1). PC1 was associated with a high scoring of “spitting”, “kicking”, and “screaming”. Consequently, PC1 was interpreted as a factor illustrating “defensive behavior”. As PC2 was associated with a high scoring of “freezing” and “collapsing” and a high plasma concentration of cortisol, the principal component was interpreted as a factor illustrating “stress”.

**Table 1:** Factor loadings of principal component (PC) 1 (defensive behavior) and PC 2 (stress).

Factors	PC1	PC2
Serum cortisol concentration	- 0.03	**0.71**
BCS	0.17	- 0.06
Leading score	0.2	0.09
Humming	- 0.11	- 0.21
Screaming	**0.55**	0.25
Snorting	0.32	0.38
Moaning	0.35	0.21
Rising	- 0.09	0
Spitting	**0.81**	- 0.06
Stomping	0.23	0.02
Kicking	**0.77**	- 0.05
Freezing	0.12	**0.6**
Collapsing	- 0.14	**0.77**

Plasma concentrations of fructosamine, glucose, total protein, and albumin as well as the PCV were tested for correlations among each other and with demographic data (sex [male, female], age [days], and body mass [kg]), parameters of the general clinical examination (heart rate, respiratory rate, body temperature [°C], and BCS), behavioral data (defensive behavior [PC1], stress behavior [PC2]), further laboratory parameters (leucocyte count, NLR, plasma cortisol, plasma globulins, and the GIN-Score) as well as the length of the transport route to the clinic [km]. Correlations of numeric variables (Table 2) were calculated using the Pearson method. Effects of sex (only two categories) were tested with Mann-Whitney U tests. Categorical parameters exceeding two categories (BCS, GIN-Score) were analyzed implementing Kruskal-Wallis tests and pairwise Wilcoxon Rank Sum tests for posthoc pairwise comparisons. To exclude age effects, tests for the parameters fructosamine and glucose were also calculated for adult animals only (from the second year of life onwards, *n* = 102).

**Table 2:** Distribution of the collected and computed numerical parameters in the investigated population of alpacas presented as patients to the veterinary clinic. PCV = packed cell volume; NLR = neutrophil-to-lymphocyte ratio.

	Unit	*n*	min	Median	max
Age	days	122	3	1288.0	6657.0
Body mass	kg	106	4.3	56.0	110.0
Respiratory rate	bpm	120	12	28.0	92.0
Heart rate	bpm	119	28	64.0	160.0
Body temperature	°C	123	35.0	38.0	39.9
Defense behavior (PC1)		102	0.0	0.9	4.8
Stress (PC2)		102	0.0	1.0	4.9
Leukocytes	10^9^/l	124	4.4	11.3	46.2
PCV	l/l	125	0.1	0.3	0.4
NLR		121	0.2	4.4	30.1
Serum cortisol	nmol/l	122	2.8	60.8	430.0
Plasma glucose	mmol/l	125	4.2	9.3	37.3
Plasma fructosamine	µmol/l	125	143.1	326.0	1037.7
Plasma total protein	g/l	123	27.2	64.9	88.7
Plasma albumin	g/l	122	15.1	38.2	48.9
Plasma globulins	g/l	122	12.1	26.9	59.4
Transportation distance	km	125	15.2	130.0	385.0

## Results

### Glucose

**Fig. 1:**
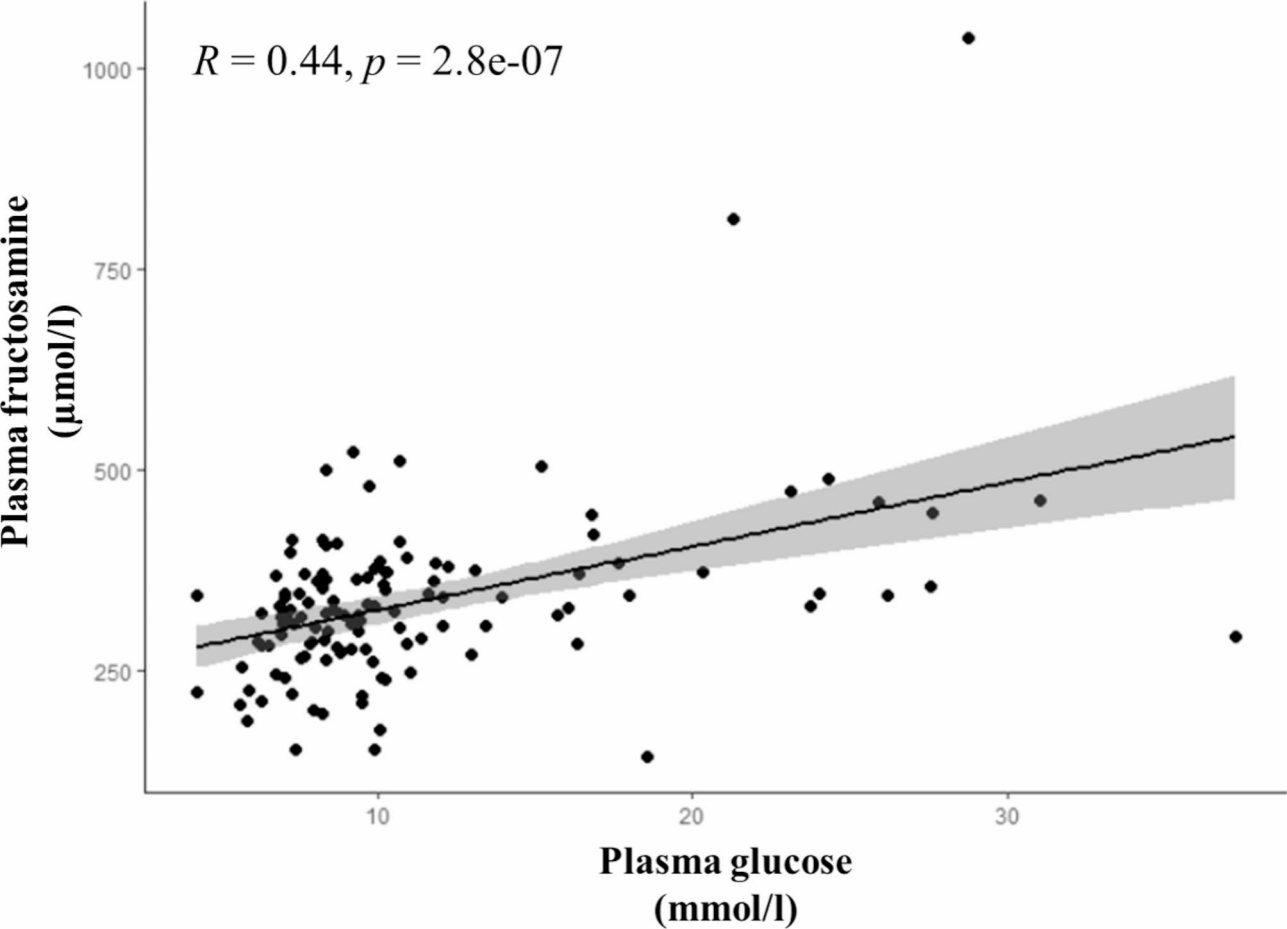
Plasma concentration of fructosamine in relation to glucose; Pearson correlation.

**Fig. 2:**
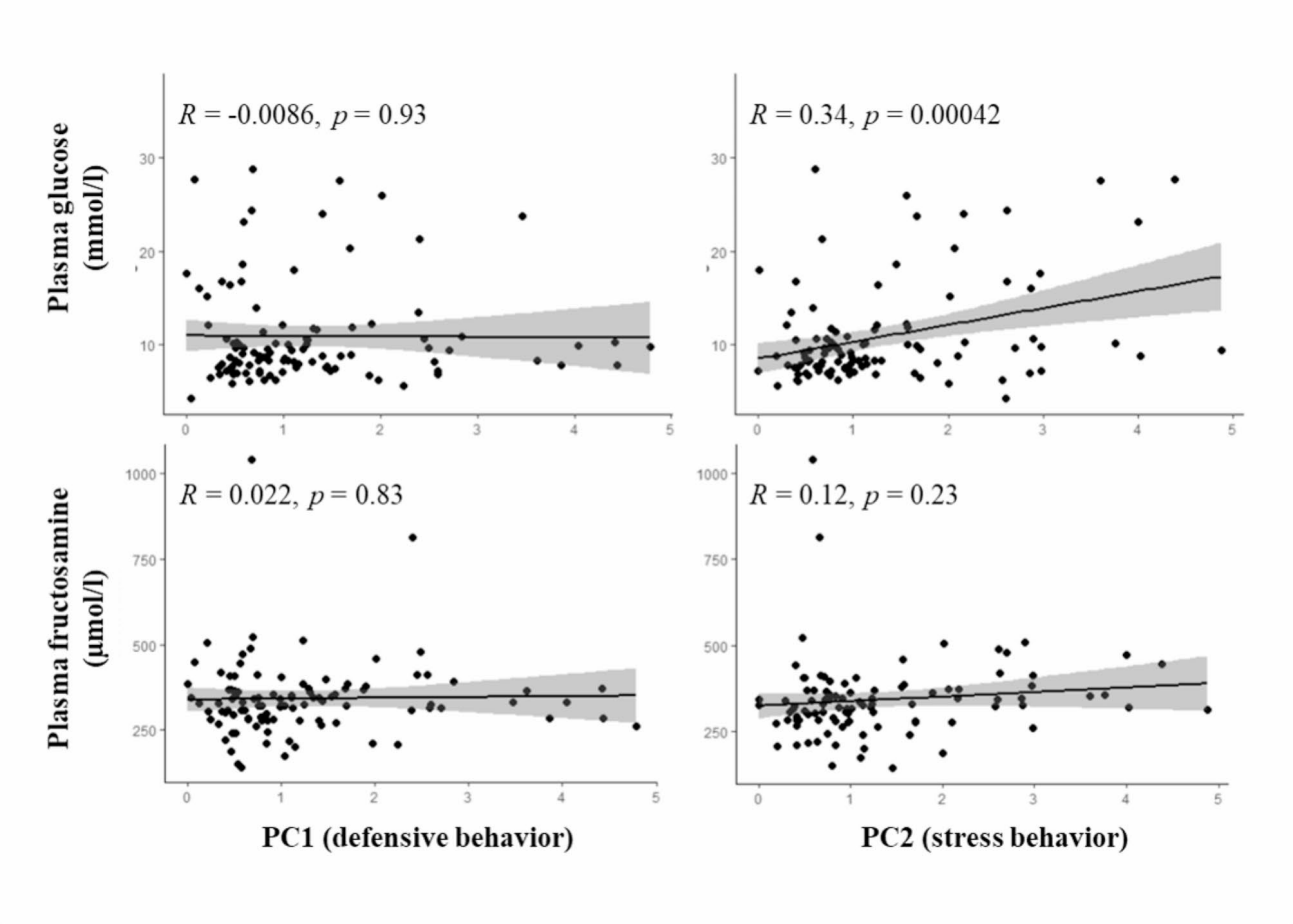
Plasma concentrations of glucose and fructosamine in relation to the two main principal components PC1 (defensive behavior) and PC2 (stress behavior); Pearson correlation.

**Fig. 3:**
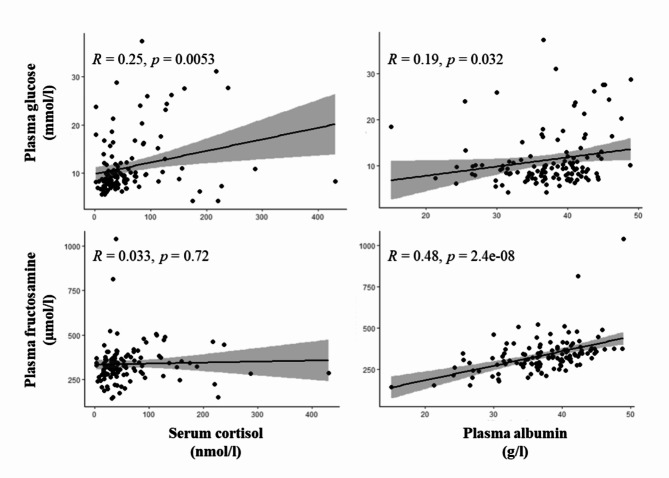
Plasma concentrations of glucose and fructosamine in relation to serum cortisol and plasma albumin; Pearson correlation.

Glucose plasma concentration was available for all 125 individuals. In relation to the reference interval of Dawson et al. (2011)^41^, two animals (1.6%) were below the lower limit of 5.5 mmol/l and 89 animals (71.2%) above the upper limit of 7.9 mmol/l. A summary of the results of the bivariate analyses is given in Table 3. In the dataset including all individuals, glucose concentration showed statistically significant positive correlations with fructosamine (*R* = 0.440, *p* < 0.001; Fig. 1), stress (PC2; *R* = 0.343, *p* < 0.001; Fig. 2), PCV (*R* = 0.284, *p* = 0.001), body mass (*R* = 0.300, *p* = 0.002), cortisol (*R* = 0.251, *p* = 0.005; Fig. 3), total protein (*R* = 0.199, *p* = 0.027), and albumin (*R* = 0.195, *p* = 0.032; Fig. 3), and a significant negative correlation with body temperature (*R* = -0.243, *p* = 0.007). By investigating only adult animals, correlations with total protein (*R* = 0.181, *p* = 0.070) and body temperature (*R* = -0.181, *p* = 0.072) were no longer significant. The remaining 11 tested parameters did not show any significant correlations.

**Table 3:** Overview of the results of the bivariate analysis of single parameter effects on the plasma glucose concentration in (A) all 125 investigated alpacas, (B) a total of 105 individuals above one year of age, and (C) 20 individuals below one year of age. Bold *p*-values indicate a significant correlation. PCV = packed cell volume; NLR = neutrophil to lymphocyte ratio; M-W-U-Test (W) = Mann-Whitney U Test with test statistic W; pears. Corr. (R) = Pearson correlation with test statistic R; K-W test (χ²) = Kruskal-Wallis Test with test statistic χ².

		Statistical test	A. Glucose total		B. Glucose adult		C. Glucose juvenile	
			Test statistic	*p*	Test statistic	*p*	Test statistic	*p*
Sex	m/f	M-W-U-Test (W)	1847.0000	0.8427	1227.5000	0.8779	45.0000	0.7664
Age	days	Pears. Corr. (R)	- 0.0011	0.9908	- 0.0705	0.4817	0.1135	0.6337
Body mass	kg	Pears. Corr. (R)	0.3001	**0.0018**	0.2130	**0.0463**	0.1596	0.5550
Respiratory rate	bpm	Pears. Corr. (R)	0.0382	0.6785	0.0727	0.4794	0.1173	0.6223
Heart rate	bpm	Pears. Corr. (R)	0.1627	0.0771	0.3154	0.0016	- 0.1095	0.6554
Body temperature	°C	Pears. Corr. (R)	- 0.2427	**0.0068**	- 0.1806	0.0721	- 0.4611	**0.0407**
BCS	0–5	K-W Test (χ²)	8.9665	0.3451	9.2663	0.2341	5.1462	0.3983
Defense behavior (PC1)		Pears. Corr. (R)	- 0.0086	0.9315	- 0.0195	0.8578	0.0933	0.7509
Stress (PC2)		Pears. Corr. (R)	0.3427	**0.0004**	0.3159	**0.0029**	0.5093	0.0629
Leukocytes	10^9/l	Pears. Corr. (R)	0.1478	0.1015	0.0797	0.4285	0.6250	**0.0032**
PCV	l/l	Pears. Corr. (R)	0.2835	**0.0014**	0.3086	**0.0016**	0.1662	0.4837
GIN-Score	0–4	K-W Test (χ²)	1.6706	0.7960	2.0846	0.7202	0.5767	0.7495
NLR		Pears. Corr. (R)	0.1697	0.0627	0.1029	0.3108	0.5660	**0.0115**
Serum cortisol	nmol/l	Pears. Corr. (R)	0.2508	**0.0053**	0.2556	**0.0103**	0.2471	0.3077
Plasma fructosamine	µmol/l	Pears. Corr. (R)	0.4400	**< 0.001**	0.4863	**< 0.001**	- 0.0262	0.9127
Plasma total protein	g/l	Pears. Corr. (R)	0.1990	**0.0274**	0.1810	0.0700	0.1684	0.4907
Plasma albumin	g/l	Pears. Corr. (R)	0.1945	**0.0319**	0.2654	**0.0076**	- 0.1705	0.4852
Plasma globulins	g/l	Pears. Corr. (R)	0.1188	0.1925	0.0322	0.7504	0.4588	**0.0482**
Transportation distance	km	Pears. Corr. (R)	0.0043	0.9621	0.0692	0.4894	- 0.3140	0.1776

### Fructosamine

Fructosamine plasma concentration was available for all 125 individuals. In relation to the reference interval of Dawson et al. (2011)^41^, 19 animals (15.2%) were below the lower limit of 252 µmol/l and 13 animals (10.4%) above the upper limit of 425 µmol/l. An overview of the results of the bivariate analyses is given in Table 4. In the dataset including all individuals, fructosamine concentration showed statistically significant positive correlations with albumin (*R* = 0.479, *p* < 0.001; Fig. 3), glucose (*R* = 0.440, *p* < 0.001; Fig. 1), total protein (*R* = 0.416, *p* < 0.001), PCV (*R* = 0.379, *p* < 0.001), globulins (*R* = 0.180, *p* = 0.0475), and body mass (*R* = 0.242, *p* = 0.012). Compared to females, male alpacas had a significantly higher fructosamine concentration (*p* = 0.021). By investigating only adult animals, the correlation with globulins (*R* = 0.066, *p* = 0.517) lost its significance. The remaining 12 tested parameters did not show any significant correlations.

**Table 4:** Overview of the results of the bivariate analysis of single parameter effects on the plasma fructosamine concentration in (A) all 125 investigated alpacas, (B) 105 individuals above one year of age, and (C) 20 individuals below one year of age. Bold *p*-values indicate a significant correlation. PCV = packed cell volume; NLR = neutrophil-to-lymphocyte ratio; M-W U test (W) = Mann-Whitney U Test with test statistic W; pears. Corr. (R) = Pearson correlation with test statistic R; K-W-Test (χ²) = Kruskal-Wallis Test with test statistic χ².

		Statistical test	A. Fructosamine total		B. Fructosamine adult		C. Fructosamine juvenile	
			Test statistic	*p*	Test statistic	*p*	Test statistic	*p*
Sex	m/f	M-W U Test (W)	1426	**0.0207**	941	**0.0349**	38	0.4119
Age	days	Pears. Corr. (R)	0.151	0.0980	0.0980	0.3269	- 0.1085	0.6490
Body mass	kg	Pears. Corr. (R)	0.242	**0.0123**	0.2492	**0.0192**	- 0.0370	0.8919
Respiratory rate	bpm	Pears. Corr. (R)	0.055	0.5477	0.1116	0.2764	0.0285	0.9050
Heart rate	bpm	Pears. Corr. (R)	- 0.102	0.2686	- 0.0040	0.9688	- 0.2669	0.2693
Body temperature	°C	Pears. Corr. (R)	- 0.098	0.2798	0.1537	0.1268	0.0063	0.9791
BCS	0–5	K-W Test (χ²)	13.997	0.0819	10.957	0.1405	3.5000	0.6234
Defense behavior (PC1)		Pears. Corr. (R)	0.022	0.8287	- 0.0340	0.7600	0.6807	**0.0074**
Stress (PC2)		Pears. Corr. (R)	0.120	0.2281	0.1228	0.2572	0.2449	0.3988
Leukocytes	10^9/l	Pears. Corr. (R)	- 0.118	0.1900	- 0.1587	0.1130	0.1516	0.5235
PCV	l/l	Pears. Corr. (R)	0.379	**< 0.001**	0.3830	**< 0.001**	0.4975	**0.0256**
GIN-Score	0–4	K-W Test (χ²)	2.3194	0.6772	4.2013	0.3795	2.3414	0.3102
NLR		Pears. Corr. (R)	0.037	0.6880	0.0233	0.8187	0.0353	0.8861
Serum cortisol	nmol/l	Pears. Corr. (R)	0.033	0.7189	0.0276	0.7852	0.0475	0.8469
Plasma glucose	mmol/l	Pears. Corr. (R)	0.440	**< 0.001**	0.4863	**< 0.001**	- 0.0262	0.9127
Plasma total protein	g/l	Pears. Corr. (R)	0.416	**< 0.001**	0.3554	**< 0.001**	0.6922	**0.0010**
Plasma albumin	g/l	Pears. Corr. (R)	0.479	**< 0.001**	0.4950	**< 0.001**	0.5449	**0.0159**
Plasma globulins	g/l	Pears. Corr. (R)	0.180	**0.0475**	0.0655	0.5172	0.6516	**0.0025**
Transportation distance	km	Pears. Corr. (R)	0.112	0.2136	0.1161	0.2452	0.2161	0.3602

### Total protein

Total protein plasma concentration was available for 123 individuals. In relation to the reference interval of Dawson et al. (2011)^41^, 26 animals (21.1%) were below the lower limit of 58 g/l and 18 animals (14.6%) above the upper limit of 73 g/l. An overview of the results of the bivariate analyses is given in Table 5. Total protein concentration showed statistically significant positive correlations with globulins (*R* = 0.802, *p* < 0.001), albumin (*R* = 0.574, *p* < 0.001), fructosamine (*R* = 0.416, *p* < 0.001), body mass (*R* = 0.402, *p* < 0.001), PCV (*R* = 0.291, *p* = 0.001), defensive behavior (PC1; *R* = 0.262, *p* = 0.008), and glucose (*R* = 0.199, *p* = 0.027), and a significant negative correlation with heart rate (*R* = -0.315, *p* < 0.001). The remaining 11 tested parameters did not show any significant correlations.

**Table 5:** Overview of the results of the bivariate analysis of single parameter effects on the plasma concentration of total protein, albumin, and the packed cell volume (PCV). Bold *p*-values indicate a significant correlation. PCV = packed cell volume; NLR = neutrophil-to-lymphocyte ratio; M-W-U-Test (W) = Mann-Whitney U Test with test statistic W; pears. Corr. (R) = Pearson correlation with test statistic R; K-W test (χ²) = Kruskal-Wallis Test with test statistic χ².

		Statistical test	Total protein *n* = 123 alpacas		Albumin *n* = 122 alpacas		PCV *n* = 125 alpacas	
			Test statistic	*p*	Test statistic	*p*	Test statistic	*p*
Sex	m/f	M-W U Test (W)	1643	0.3230	1771.0000	0.8820	1418.5	**0.0185**
Age	days	Pears. Corr. (R)	0.176	0.0541	- 0.2321	**0.0111**	- 0.073	0.4244
Body mass	kg	Pears. Corr. (R)	0.402	**< 0.001**	0.1208	0.2242	0.184	0.0591
Respiratory rate	bpm	Pears. Corr. (R)	- 0.012	0.8954	0.1669	0.0720	0.055	0.5477
Heart rate	bpm	Pears. Corr. (R)	- 0.315	**0.0005**	- 0.1727	0.0638	- 0.102	0.2686
Body temperature	°C	Pears. Corr. (R)	- 0.015	0.8699	- 0.0002	0.9985	0.098	0.2798
BCS	0–5	K-W Test (χ²)	14.822	0.0627	36.7700	**< 0.001**	20.182	**0.0097**
Defense behavior (PC1)		Pears. Corr. (R)	0.262	**0.0082**	- 0.0140	0.8901	- 0.066	0.5089
Stress (PC2)		Pears. Corr. (R)	0.095	0.3451	0.1930	0.0544	0.296	**0.0025**
Leukocytes	10^9/l	Pears. Corr. (R)	0.026	0.7804	- 0.0831	0.3650	- 0.158	0.0791
PCV	l/l	Pears. Corr. (R)	0.291	**0.0011**	0.4888	**< 0.001**		
GIN-Score	0–4	K-W Test (χ²)	3.9293	0.4157	8.8961	0.0638	14.879	**0.0050**
NLR		Pears. Corr. (R)	0.048	0.6031	- 0.0008	0.9935	- 0.064	0.4840
Serum cortisol	nmol/l	Pears. Corr. (R)	- 0.074	0.4248	- 0.1117	0.2266	0.263	**0.0035**
Plasma glucose	mmol/l	Pears. Corr. (R)	0.199	**0.0274**	0.1945	**0.0319**	0.284	**0.0014**
Plasma fructosamine	µmol/l	Pears. Corr. (R)	0.416	**< 0.001**	0.4788	**< 0.001**	0.379	**< 0.001**
Plasma total protein	g/l	Pears. Corr. (R)			0.5744	**< 0.001**	0.291	**0.0011**
Plasma albumin	g/l	Pears. Corr. (R)	0.574	**< 0.001**			0.489	**< 0.001**
Plasma globulins	g/l	Pears. Corr. (R)	0.802	**< 0.001**	- 0.0282	0.7575	0.030	0.7447
Transportation distance	km	Pears. Corr. (R)	- 0.049	0.5901	0.0880	0.3300	0.056	0.5365

### Albumin

Albumin plasma concentration was available for 122 individuals (Table 5). In relation to the reference interval of Dawson et al. (2011)^41^, 11 animals (9.0%) were below the lower limit of 28 g/l and 14 animals (11.5%) above the upper limit of 43 g/l. Albumin showed significant positive correlations with total protein (*R* = 0.574, *p* < 0.001), PCV (*R* = 0.489, *p* < 0.001), fructosamine (*R* = 0.479, *p* < 0.001; Fig. 3), and glucose (*R* = 0.195, *p* = 0.032; Fig. 3), and a significant negative correlation with age (*R* = 0.232, *p* = 0.011. Compared to a BCS of 1 and 1.5, animals with a BCS of 3, 3.5, and 4 showed significantly higher plasma albumin concentrations (*p* < 0.001). The remaining 11 tested parameters did not show any significant correlations.

### Packed cell volume

PCV was available for all 125 individuals (Table 5). In relation to the reference interval of Dawson et al. (2011)^50^, 17 animals (13.6%) were below the lower limit of 0.22 l/l. No animal exceeded the upper limit of 0.45 l/l. PCV showed significant positive correlations with albumin (*R* = 0.489, *p* < 0.001), fructosamine (*R* = 0.379, *p* < 0.001), total protein (*R* = 0.291, *p* = 0.001), glucose (*R* = 0.284, *p* = 0.001), stress (PC2; *R* = 0.296, *p* = 0.003) and cortisol (*R* = 0.263, *p* = 0.004). Compared to animals with high-grade gastrointestinal nematode infections, animals with no shedding of nematode eggs showed significantly higher PCV values (*p* = 0.005). Compared to females, PCV in male alpacas was significantly higher (*p* = 0.018). The remaining 10 tested parameters did not show any significant correlations.

## Discussion

**Fig. 4:**
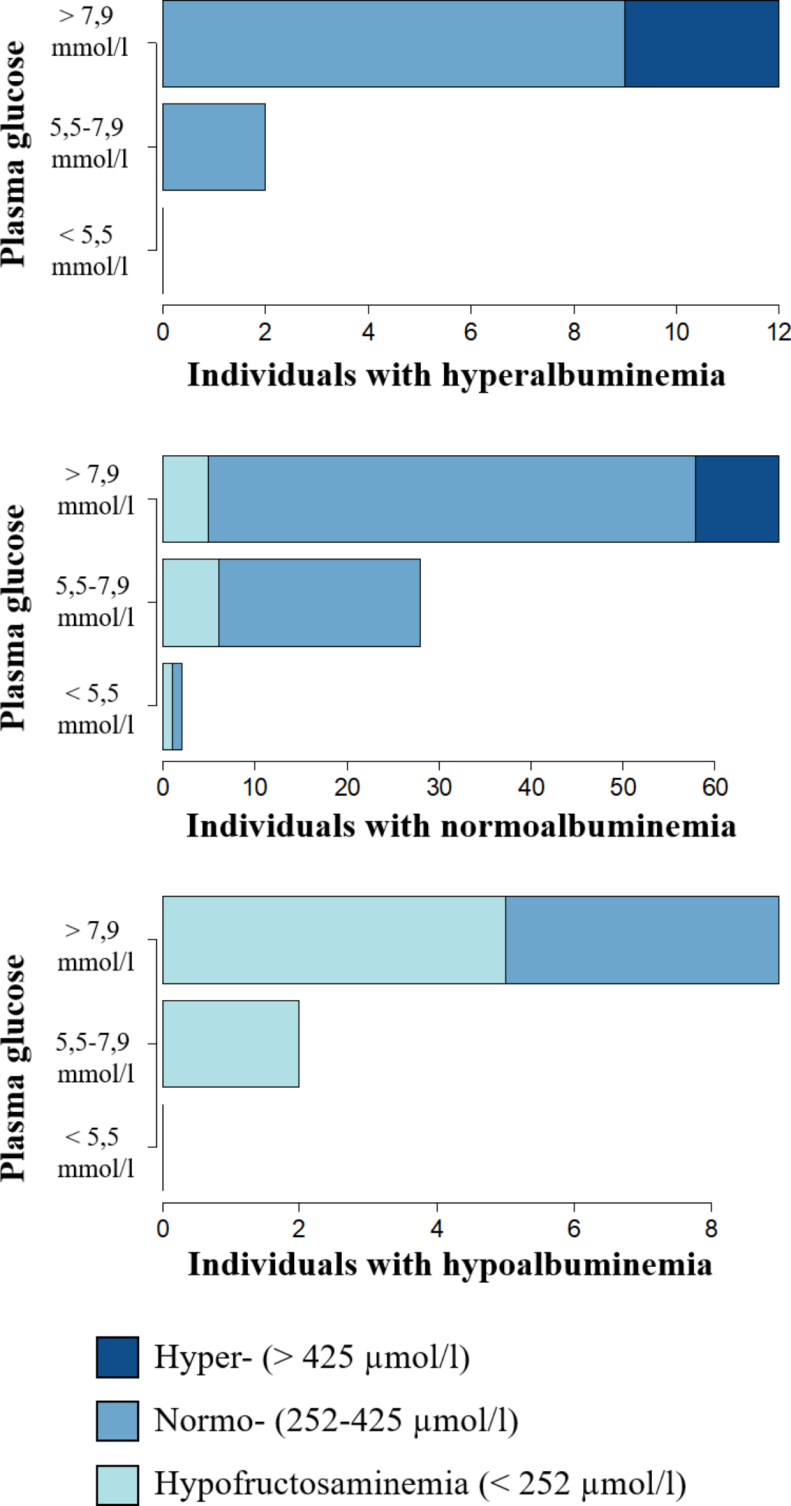
Plasma fructosamine concentration (blue shades) in relation to plasma glucose concentration (Y-axis of the bar plots) divided according to plasma albumin concentration.

On account of the peculiarities of the carbohydrate metabolism of camelids, it was expected to find a large proportion of hyperglycemia among the studied alpacas (Fig. 4). With 71.2%, more than two thirds of the animals exceeded the reference interval for plasma glucose. As a significant correlation of stress behavior and serum cortisol concentration with plasma glucose concentration could be demonstrated, stress due to pain, symptoms of disease, transportation, medical examination, and the unfamiliar environment seems to be the most probable reason for this observation.

Serum cortisol was used in this study as an indicator for stress. Nevertheless, an individual basal value is necessary to evaluate a cortisol concentration, which was not available here. Further treatment and sampling of the patients varied greatly from individual to individual, comparable follow-up examinations were hence not possible. In a study on cortisol response of alpacas to a stressful event, a mean serum cortisol baseline of 20.7 nmol/l was determined in 12 individuals, compared to mean cortisol levels of 49.3 nmol/l after short distance transportation^56^. As the individual values hardly deviated from the mean (standard deviations of a maximum of 5 nmol/l), a comparison with the data from our study might, however, be possible. These basal values determined by Anderson and colleagues^56^ were usually greatly exceeded by the animals we examined: only 22 individuals showed serum cortisol concentrations below 20 nmol/l, 55 showed concentrations between 20 and 50 nmol/l, 26 showed concentrations between 50 and 100 nmol/l, and 19 animals showed even concentrations of more than 100 nmol/l with a maximum of 430 nmol/l. The informative value of a single cortisol measurement as a stress indicator was additionally confirmed by the principal component analysis, where stress-associated behaviors clustered with cortisol concentration. In addition to the association of glucose with stress, a positive correlation with body mass, PCV, total protein and albumin could be demonstrated. This may be related to the availability of glucose through adequate dietary intake in animals in good body condition. A reverse effect appeared to have been largely masked by stress hyperglycemia. Hypoglycemia was almost absent and only observed in two individuals (both male; one six months old, one 10 years old).

In contrast to the plasma glucose concentration, most of the examined individuals (93/125, 74.4%) showed plasma fructosamine concentrations within the reference interval of 252–425 µmol/l published by Dawson et al. (2011)^41^. Fructosamine therefore appears to be largely unaffected by the acute stress event of transportation and examination in our clinic. This conclusion is also supported by the absence of correlations of plasma fructosamine concentration with stress behavior and serum cortisol. Whether chronic stress can lead to chronic hyperglycemia and thus to an increased plasma fructosamine concentration is not entirely clear. However, this should be considered in any case, as clinical diseases caused by chronic stress in SAC are well documented^3,4,7^ and the physiological dependencies of the individual factors can be, looking on the present data, strongly assumed. Fructosamine concentrations below the reference interval were observed in 19 animals (15.2%). Of these, 17 showed hypoproteinemia and seven simultaneously hypoalbuminemia. Hypoglycemia was observed in only one of these animals, while eight showed glucose concentrations in the reference range and 10 were even hyperglycemic. Low fructosamine concentrations therefore appear to be mainly mediated by the availability of the protein component. In addition to the correlation with glucose (*R* = 0.440, *p* < 0.001), this can be confirmed by the strong positive correlation of fructosamine concentration with albumin (*R* = 0.479, *p* < 0.001), globulins (*R* = 0.180, *p* = 0.048), and hence also total protein (*R* = 0.416, *p* < 0.001). Globulins, however, appear to have a particular relevance in young animals. Although a significant correlation was found in the overall data set, this could no longer be found when animals over one year of age (*n* = 105) were examined separately but was found in animals under one year of age (*n* = 20). This observation can certainly be explained by the relative decrease in the globulin fraction and increase in the albumin fraction as the animals mature^57^. Acute-phase proteins as part of the total protein fraction were not individually tested for their relationship to fructosamine. Whether these proteins play a role in the development of fructosamine in alpacas unfortunately cannot be clarified here. Overall, not much is known about the measurement and importance of acute-phase proteins in alpacas^58^. It is therefore certainly worth investigating their relationship to fructosamine when more information about them is available for alpacas. In 13 animals with plasma fructosamine concentrations above the reference range (13/125, 10.4%), a permanent disturbance of the carbohydrate metabolism could be assumed; all 13 animals showed hyperglycemia. Whether an excessive availability of protein could also be a reason is difficult to assess. A percentage of 23% of the 13 animals (3/13) showed hyperproteinemia, mostly in conjunction with hyperalbuminemia. Looking at the entire study population, the proportion of animals with hyperproteinemia (14%, 18/123) was at a similar level. In most cases of hyperproteinemia, not an absolute but a relative increase of plasma protein in the context of dehydration should be involved. This is simultaneously associated with a relative increase in PCV, which in turn correlates significantly positive with the plasma fructosamine concentration. PCV could, however, also be affected without any alteration in plasma protein concentration. In analogy to infections with hemotrophic mycoplasmas in other species, it could be speculated that SACs that are infected with *Candidatus* Mycoplasma haemolamae could also suffer from a decreased PCV due to the death of infected erythrocytes^59^. Nevertheless, studies by Tornquist et al. (2010)^60^, Viesselmann et al. (2019)^61^ and Wagener et al. (2024)^62^ found no association between anemia and an infection with *Candidatus* Mycoplasma haemolamae in alpacas or llamas. This was even the case when patients from our clinic with underlying disease were examined^62^. However, due to the strong positive correlation between PCV and total protein and albumin, this effect is probably negligible. In the available data set, only a few individual animals have been examined for infections with this blood parasite. Also, the positive correlation between fructosamine and body mass is most likely mediated by the dependence of fructosamine on proteins. Hypoproteinemia in SACs is mostly induced by endoparasite infections, particularly with gastrointestinal nematodes^46,63–65^. In addition to hypoproteinemia, severe infections, especially with the nematode species *Haemonchus contortus*, are associated with a drop in packed cell volume and a decline in body condition^66,67^. This could also be shown directly by a significant positive correlation of total protein and albumin concentration with PCV, and total protein concentration with body mass. In addition, significantly higher albumin concentrations were shown in animals with good body condition (BCS of 3.0, 3.5, and 4.0) compared to emaciated animals with a low BCS (1.0 and 1.5). A direct relationship to parasitological diagnostics was only found for PCV, where animals without shedding of gastrointestinal nematode eggs showed significantly higher PCV values than animals with high-grade shedding. Fructosamine plasma concentration was additionally found to be significantly higher in males than in female alpacas. A similar observation was also made by Dawson et al. (2011)^41^ in their dataset of 74 clinically healthy alpacas. Sex-related differences were also found in other species like cats^68^, Rhesus macaques^69^, and silver foxes^70^, where always male individuals showed higher fructosamine concentrations. Whether protein availability is responsible for this observation is not entirely clear. Protein concentrations in Rhesus macaques were not investigated; in cats, no differences in protein concentrations were found between sexes; male silver foxes, however, showed higher concentrations of β-globulins and male alpacas were found to have higher γ-globulin concentrations than females^41^. In the present study, no sex-related differences in total protein and albumin concentration were found. Nevertheless, the single globulin fractions were not examined individually.

## Conclusions

The study was able to show that high plasma glucose concentrations occurred together with high serum cortisol levels and certain stress-associated behaviors (“collapsing” and “freezing”). Acutely stressed animals can therefore particularly be recognized by their stuporous behavior - collapsed and completely motionless. Overall, the measurement of plasma fructosamine in alpacas allows to identify single individuals with possibly permanently high glucose plasma concentration levels from the vast majority of animals with acute hyperglycemia caused by the stressful situation of transport and clinical examination. In addition, as already described in other animal species, the availability of protein (particularly albumin) also appears to have a major impact on plasma fructosamine concentration in alpacas. In particular and to prevent false negative results, total protein or albumin plasma concentration should be taken into account when evaluating laboratory results. If the interpretation of measured fructosamine concentration leads to the suspicion of chronic hyperglycemia, a diabetic like syndrome or already chronic stress might be responsible for rising fructosamine concentrations. However, the parameter alone is not capable of differentiating the type of hyperglycemia. Nevertheless, fructosamine might be a valuable parameter in routine diagnostics to distinguish acutely stressed from chronically affected patients.

## Electronic supplementary material

Below is the link to the electronic supplementary material.

Supplementary Material 1

## Data Availability

Data are available upon request from the corresponding author.
